# Associations of sleep-associated variants with wearable-derived sleep stages in the *All of Us* research program

**DOI:** 10.1093/sleep/zsaf209

**Published:** 2025-07-26

**Authors:** Irene N Chan, Seyed Mehdi Nouraie, Stephen Y Chan, Neil J Kelly

**Affiliations:** Center for Pulmonary Vascular Biology and Medicine, Pittsburgh Heart, Lung, and Blood Vascular Medicine Institute, Department of Medicine, University of Pittsburgh School of Medicine and University of Pittsburgh Medical Center, Pittsburgh, PA 15213, USA; Heart and Vascular Institute, Department of Medicine, University of Pittsburgh School of Medicine and University of Pittsburgh Medical Center, Pittsburgh, PA 15213, USA; Center for Pulmonary Vascular Biology and Medicine, Pittsburgh Heart, Lung, and Blood Vascular Medicine Institute, Department of Medicine, University of Pittsburgh School of Medicine and University of Pittsburgh Medical Center, Pittsburgh, PA 15213, USA; Division of Pulmonary, Allergy, and Critical Care Medicine, Department of Medicine, University of Pittsburgh School of Medicine and University of Pittsburgh Medical Center, Pittsburgh, PA 15213, USA; Center for Pulmonary Vascular Biology and Medicine, Pittsburgh Heart, Lung, and Blood Vascular Medicine Institute, Department of Medicine, University of Pittsburgh School of Medicine and University of Pittsburgh Medical Center, Pittsburgh, PA 15213, USA; Heart and Vascular Institute, Department of Medicine, University of Pittsburgh School of Medicine and University of Pittsburgh Medical Center, Pittsburgh, PA 15213, USA; Center for Pulmonary Vascular Biology and Medicine, Pittsburgh Heart, Lung, and Blood Vascular Medicine Institute, Department of Medicine, University of Pittsburgh School of Medicine and University of Pittsburgh Medical Center, Pittsburgh, PA 15213, USA; Heart and Vascular Institute, Department of Medicine, University of Pittsburgh School of Medicine and University of Pittsburgh Medical Center, Pittsburgh, PA 15213, USA; Division of Cardiology, Department of Medicine, VA Pittsburgh Healthcare System, Pittsburgh, PA 15240, USA

## 1. Introduction

Sleep is an essential yet incompletely understood process regulated by environmental, homeostatic, and genomic cues.^1^ Despite substantial evidence linking insufficient sleep quantity to chronic diseases and all-cause mortality,^2^^,^^3^ recent estimates suggest that more than one-third of adults in the United States fail to achieve the recommended 7 hours of daily sleep.^4^ Hence, strengthening our understanding of the factors influencing sleep duration is of critical public health importance.

American Academy of Sleep Medicine guidelines^5^ divide sleep into five stages, including wakefulness, three stages of non-rapid eye movement (NREM) sleep (N1-N3), and rapid eye movement (REM) sleep. Each sleep stage is thought to serve essential physiologic functions,^1^ with a recent study suggesting that deficiencies in various sleep stages are associated with unique disease spectra.^2^

In the past 2 decades, genome-wide association studies have identified hundreds of loci associated with objective and self-reported sleep duration.^6^ However, the impacts of these genomic variants on sleep stages are not defined. Knowledge of these associations may lead to greater understanding of the roles of specific sleep stages in human health.

With the advent of commercial-grade biosensors such as Fitbit, sleep stage estimates based on accelerometry and photoplethysmography have become widely available.^2^ The *All of Us* (*AoU*) Research Program contains Fitbit-derived sleep stage estimates of light (N1 + N2), deep (N3), and REM sleep, accompanied by whole-genome sequencing.^7^ Using this resource, we hypothesized that genomic variants with reported associations to sleep duration have distinct associations to constituent stages of human sleep.

## 2. Methods

### 2.1. Data availability

*AoU* data and code are available to authorized users of the *AoU* Research Program’s Controlled Tier Dataset 8 on the Researcher Workbench at https://www.researchallofus.org/.

### 2.2. All of Us

The study period encompassed January 1, 2018, through October 1, 2023. Participants with at least 180 days of sleep data were included based on a prior study, and for improved control of seasonal variations in sleep duration^2^^,^^8^; those with sex chromosome ploidy other than XX or XY were excluded.

### 2.3. Sleep stages

Estimates of daily light, deep, and REM sleep were obtained from the *AoU* workbench, with total sleep as their sum.

### 2.4. Single nucleotide variants

Single nucleotide variants (SNVs) associated with sleep duration (*N* = 868 in *AoU* genomics records) were obtained from the Genome Wide Association Study (GWAS) Catalog^9^ (trait identifier EFO_0005271). Genotypes, sex ploidy, and genomic ancestry were obtained from the *AoU* workbench.^7^ Allele frequencies (AFs) were calculated for each SNV allele based on frequencies in the study population (*N* = 15 111). For each SNV, the two most common alleles were studied; the reference allele was defined as the most frequent allele, and the alternate as the second most frequent. 73 SNVs with alternate allele AF < 1 per cent and one SNV with genotype calls consisting of the two studied alleles in less than 20 participants—per *AoU* policy—were excluded, with 794 SNVs remaining in the analysis (Table S1).

### 2.5. Genotype-phenotype association

Average durations of daily sleep parameters (total, light, deep, and REM) were modeled as a function of genotype, age, sex ploidy, and the first three principal components of predicted genomic ancestry using ordinary least squares regression with robust standard errors in *statsmodels* v0.14.2.^10^ Genotypes were modeled additively as the number of copies of the alternate allele. Participants whose SNV genotype included alleles other than the two most common alleles were excluded from the analysis of that SNV. Age was computed as the mean age on dates for which sleep data was available. Benjamini–Hochberg family-wise error-rate-adjusted *p*-values were computed with *scipy* v1.11.2.^11^

### 2.6. Sensitivity analyses

Sensitivity analyses were conducted to examine the effect of weekends, weekdays, and sleep timing on the identified genomic associations. Weeknights were defined as sleep periods ending on Monday through Friday; weekend nights were defined as sleep periods ending on Saturday or Sunday. Typical sleep periods were defined as those with both sleep onset time within the 08:00 pm–04:00 am interval and wake time within the 04:00 am–12:00 pm interval^12^; episodes not meeting these criteria were considered atypical.

### 2.7. *Statistic*s

Summary statistics were plotted in GraphPad Prism v10.2.0 for Windows (GraphPad Software, Boston, MA). Statistical tests were performed in Python v3.10.12. Comparisons of sleep duration between categories were made by two-sided independent *t*-test (2 groups) or one-way Analysis of Variance (ANOVA) with Holm–Bonferroni adjustment for family-wise error rate (three or more groups) using the Python packages *scipy* and *scikit-posthocs* v0.11.2. Adjusted *p*-value < .05 was considered statistically significant.

## 3. Results

### 3.1. Sleep architecture in the AoU Research Program

The controlled data repository in Version 8 of the *AoU* Research Program contains data on 633 547 participants. Among 51 249, *AoU* participants with linked wearable data during the study period, 36 915 provided whole genome sequencing and had either XX or XY sex chromosome ploidy. In line with prior studies,^2^ we limited our analyses to participants with greater than 180 days of sleep data (*N* = 15 111, Figure S1). Participants provided sleep data from a median of 685 dates (interquartile range [IQR] 384, 1168). Daily average total, light, deep, and REM sleep were normally distributed in the study population (Figure S2, A–D) and their durations were consistent with prior reports.^2^

Participants had a median age of 55 years [IQR 40–67]; total, deep, and REM sleep declined, and light sleep increased with age, consistent with prior literature.^13^ Sex chromosome ploidy was 70.61 per cent XX, and XX participants had significantly greater durations of total, deep, and REM sleep, in line with published studies.^14^ Ancestral composition was predominantly European, and participants with African genomic ancestry had the shortest duration of total, light, and REM sleep. Relationships between genomic ancestry and sleep duration are not well-established, although these results reflect observations from race-based studies^15^ (Table 1). Taken together, the background characteristics suggest the integrity of the Fitbit-derived sleep stage estimates in the *AoU* dataset.

**Table 1:** Baseline Participant Characteristics

		**Total (min.)**	**Light (min.)**	**Deep (min.)**	**REM (min.)**
All Participants	15 111	397.47 ± 46.26	256.87 ± 36.66	59.91 ± 14.72	80.69 ± 19.11
Age (years)	55 [40, 67]				
<40	3712 (24.56)	408.34 ± 40.78	248.77 ± 31.98	70.64 ± 12.86	88.94 ± 16.49
40–65	7021 (46.46)	394.07 ± 46.46^*^	252.60 ± 35.73^*,†^	59.81 ± 13.11^*,†^	81.65 ± 18.13^*,†^
>65	4378 (28.97)	393.71 ± 48.80^*^	270.59 ± 38.11^*^	50.97 ± 12.43^*^	72.16 ± 19.25^*^
Sex ploidy					
XX	10 670 (70.61)	402.70 ± 45.23	257.05 ± 35.56	61.40 ± 14.08	84.25 ± 18.10
XY	4441 (29.39)	384.92 ± 46.29^‡^	256.46 ± 39.19	56.32 ± 15.57^‡^	72.14 ± 18.76^‡^
Ancestry prediction					
AFR	907 (6.00)	367.64 ± 46.17	228.47 ± 34.09	63.12 ± 13.39	76.05 ± 17.46
AMR	1041 (6.89)	392.43 ± 44.18^§,||,¶^	244.66 ± 33.62^§,||,¶,**^	64.52 ± 14.21^¶^	83.25 ± 18.57^§,¶^
EAS	366 (2.42)	380.45 ± 40.34^§,¶^	236.69 ± 32.62^§,¶^	62.39 ± 13.92^¶^	81.37 ± 16.96^§^
EUR	12 549 (83.05)	400.75 ± 45.75^§,**^	260.88 ± 35.88^§,**^	59.11 ± 14.74^§,#,**^	80.76 ± 19.32^§^
MID	22 (0.15)	396.70 ± 46.44^§^	248.33 ± 34.39^§^	68.68 ± 15.64	79.69 ± 14.63
SAS	226 (1.50)	386.24 ± 39.94^§^	238.14 ± 29.46^§^	65.41 ± 14.87	82.69 ± 16.58^§^

Data are *n* (%), median [IQR], or mean ± standard deviation. Statistical comparisons were made by two-sided independent *t*-test (sex ploidy) or one-way ANOVA with Holm–Bonferroni adjustment for family-wise error rate (age, ancestry prediction). AFR: African; AMR: Ad Mixed American; EAS: East Asian; EUR: European; MID: Middle Eastern; SAS: South Asian. Superscripts indicate *p* < .05 as compared to under 40 (*), over 65 (†), XX (‡), AFR (§), EAS (||) EUR (¶), MID (#), or SAS (**).

*Genomic associations of sleep duration-related SNVs with sleep stages*

From the GWAS catalog,^9^ we identified 794 SNVs (minor AF of at least 1 per cent)^16^ with reported associations to sleep duration. We tested the association of the two most frequent alleles of each SNV with the mean daily durations of total, light, deep, and REM sleep adjusted for age, genomic ancestry, and sex chromosome ploidy in a multiple linear regression model.

**Figure 1:**
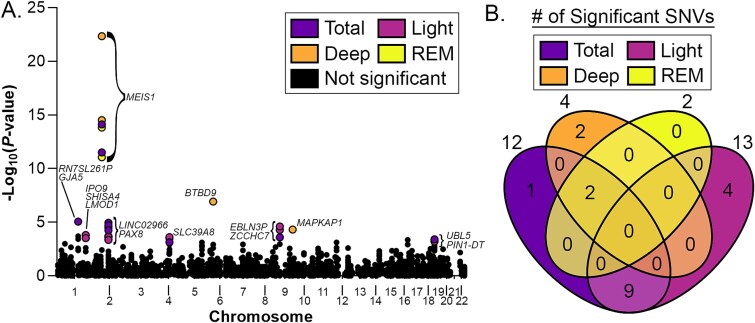
Identification of SNVs associated with sleep architecture. (A) Manhattan plot of SNV associations with total, light, deep, and REM sleep, color coded by sleep phenotype. (B) Venn diagram indicates the number of significant SNVs for total, light, deep, and REM sleep.

We identified 12, 13, 4, and 2 SNVs associated with total, light, deep, and REM sleep duration, respectively (Figure 1, Tables S2–S6). For each significant association, the same allelic variant was associated with shorter sleep stage duration—albeit to varying degrees—and the allelic directionality of change was consistent with prior reports.^17–22^ As expected, the majority of SNVs (11 of 12) associated with total sleep duration were also associated with the lengths of other sleep stages, and total sleep duration had overlapping associations with each stage examined. In contrast, of the 13 SNVs associated with light sleep duration, none overlapped with deep or REM sleep duration. Meanwhile, deep and REM sleep had partially overlapping SNV associations. SNVs associated with light sleep duration corresponded to five genomic loci mapping to the genes *IPO9*, *SHISA4*, *LMOD1*, *PAX8*, *LINC02966*, *SLC39A8*, *EBLN3P*, *ZCCH7*, *UBL5*, and *PIN1-DT*. The three loci linked to deep sleep mapped to genes *MEIS1*, *BTBD9*, and *MAPKAP1*, while the locus linked to REM sleep mapped to *MEIS1*.

We next performed sensitivity analyses of sleep periods averaged across weekdays or weekends (Table S7, Figure S3), atypical or typical sleep periods (Table S8, Figure S4), and averaged across all sleep periods in the participant subsets with up to or greater than 90 per cent typical sleep periods (Table S9, Figure S5). In analyses of weekdays, weekends, typical sleep periods, and participants with greater than 90 per cent typical sleep periods, we saw consistent associations between total sleep duration and *MEIS1*, *LINC02966*, and *PAX8*-associated variants; between deep sleep duration and *MEIS1* and *BTBD9*-associated variants; and between REM sleep duration and *MEIS1*-associated variants. Collectively, these findings are consistent with the idea of distinct pathways preferentially influencing different elements of sleep architecture.

## 4. Discussion

In this study, we identified novel associations between wearable-derived sleep stages and genomic variants related to sleep duration. To our knowledge, this is the first study to report genomic associations with specific sleep stages and suggests that these stages may be controlled by distinct genomic influences.

By design, our study was limited to variants previously associated with sleep duration in order to define their relationships to sleep stages.^9^ Many of the variants investigated were not associated with total sleep duration in our analyses, which differed from prior studies in key respects. We studied sleep duration as a continuous variable from longitudinal objective recordings, whereas many prior studies have been limited to extreme phenotypes (e.g. short versus long sleepers) or self-reported sleep duration. Additionally, our study included an ancestrally-diverse cohort, while rare previous reports have extended beyond Europeans.^17^^,^^22^ Under this framework, we identified 12 SNVs associated with total sleep duration as well as SNVs associated with light, deep, and REM sleep. Notably, the strongest associations with total sleep identified in this study align with previous wearable-based reports.^19^^,^^23^

Our major finding is the association of separate sets of variants with different sleep stages. While we identified the greatest number of SNVs, 13, associated with light sleep duration, we also identified four and two SNVs associated with deep and REM sleep duration, respectively. Among these, rs113851554 and rs11693221 (mapped to the *MEIS1* gene) were associated with both deep and REM sleep duration, while rs9369062 (mapped to the *BTBD9* gene) was associated with deep sleep only. It is possible that these variants also impact light sleep duration, albeit to a lesser magnitude, which our study was underpowered to detect. These variants have previously been associated with both sleep duration^23^ and restless leg syndrome (RLS).^24^ Importantly, RLS is not thought to affect the accuracy of accelerometer-derived sleep estimates,^23^ although it is unknown whether that extends to sleep stages. Polysomnographic studies have demonstrated that RLS preferentially affects later sleep stages,^25^ and our results add genomic support to this conclusion. *MEIS1* encodes a homeobox transcription factor involved in hematopoiesis and neurodevelopment, while *BTBD9* encodes a BTB(POZ) domain protein highly expressed in brain; both have been experimentally linked to iron homeostasis,^26^^,^^27^ although it is unclear whether their variant associations to deep sleep are exerted via (in)direct actions or because of the clinical syndrome of RLS. Additionally, we found a significant association between rs2416963, located near the *MAPKAP1* gene, which encodes a subunit of mammalian Target of Rapamycin Complex 2 (mTORC2), and deep sleep. This gene locus, which was previously linked to device-measured sleep duration,^19^ has no reported association to RLS and may represent a novel mediator of deep sleep duration.

We acknowledge limitations to this work. While polysomnography remains the gold-standard in sleep stage determination,^28^ Fitbit-derived estimates offer a feasible means of studying longitudinal sleep stage patterns in large populations. Meanwhile, Fitbit has additional advantages over traditional self-reported sleep estimates or sleep diaries, both of which are unable to capture the constituent stages of sleep. Second, our analyses may be impacted by unmeasured confounding variables, such as socioeconomic status, use of sleep-altering medications, and exposure to solar irradiation. Third, our study focused only on common and low-frequency SNVs that had been previously linked to total sleep duration, potentially overlooking novel variants that contribute only to specific elements of sleep architecture. Additionally, many participants were excluded due to the requirement for 180 nights of sleep data. These findings require validation in independent cohorts, particularly given that the *AoU* Fitbit population has predominantly XX sex chromosome ploidy and may otherwise not reflect the general population.

In summary, our study identifies variants associated with sleep stages as determined by Fitbit wearables. The finding of stage-specific SNV associations highlights that variant links to sleep duration may have very different biological meanings depending on the stage affected.^2^ If confirmed, these findings may provide a foundation for identification of specific pathways and gene networks regulating the various constituent stages of human sleep.

## Supplementary Material

Chan_et_al_2025_Supplemental_Figures_zsaf209

Chan_et_al_2025_Supplemental_Tables_zsaf209
