# Supplementary material for: Associations of sleep-associated variants with wearable-derived sleep stages in the All of Us research program
Source: Sleep. 2025 Jul 26;48(11):zsaf209. doi: 10.1093/sleep/zsaf209 (PMC12597665; doi:10.1093/sleep/zsaf209)

**ONLINE DATA SUPPLEMENT**

**Associations of sleep-associated variants with wearable-derived sleep stages in the *All of Us* research program.**

Irene N. Chan^1,2^, Seyed Mehdi Nouraie^1,3^, Stephen Y. Chan^1,2,*^, and Neil J. Kelly^1,2,4,*^

^1^Center for Pulmonary Vascular Biology and Medicine, Pittsburgh Heart, Lung, and Blood Vascular Medicine Institute, ^2^Heart and Vascular Institute, ^3^Division of Pulmonary, Allergy, and Critical Care Medicine, Department of Medicine, University of Pittsburgh School of Medicine and University of Pittsburgh Medical Center, Pittsburgh, PA 15213, USA. ^4^VA Pittsburgh Healthcare System, Pittsburgh, PA 15240, USA.

**^*^Corresponding Authors:**

Stephen Y. Chan, MD, PhD

Pittsburgh Heart, Lung, and Blood Vascular Medicine Institute

University of Pittsburgh School of Medicine and UPMC

E1240 Biomedical Science Tower

200 Lothrop Street

Pittsburgh, PA 15261

Phone: (412) 383-6990

Email: [chansy@pitt.edu](mailto:chansy@pitt.edu)

Neil J. Kelly, MD, PhD (**contact**)

Pittsburgh Heart, Lung, and Blood Vascular Medicine Institute

University of Pittsburgh School of Medicine and UPMC

1702 Biomedical Science Tower

200 Lothrop Street

Pittsburgh, PA 15261

Phone: (412) 383-6030

Email: [njk88@pitt.edu](mailto:njk88@pitt.edu)

**SUPPLEMENTAL FIGURE CAPTIONS**

**Figure S1**. *Flow diagram of participant selection.*

**Figure S2**. *Histograms and distributions of sleep stage duration.* Histograms of sleep stage were generated for (**A**) total, (**B**) light, (**C**) deep, and (**D**) REM sleep duration (N = 15,111). Adjacent bins were combined such that each bin contained data from at least 20 participants. Red dashed curve represents Gaussian distribution. Vertical black dashed line represents population mean.

**Figure S3.** *Identification of SNVs associated with weekday and weekend sleep architecture.* Mean sleep stage duration was aggregated over all sleep periods ending on a weekday or weekend. (**A**) Manhattan plot of SNV associations with total, light, deep, and REM sleep, color coded by sleep phenotype and (**B**) Venn-diagram indicating the number of significant SNVs for total, light, deep, and REM sleep across weekday sleep periods. (**C**) Manhattan plot of SNV associations with total, light, deep, and REM sleep, color coded by sleep phenotype and (**D**) Venn-diagram indicating the number of significant SNVs for total, light, deep, and REM sleep across weekend sleep periods.

**Figure S4.** *Identification of SNVs associated with sleep architecture across atypical and typical sleep periods.* Mean sleep stage duration was aggregated over all atypical or typical sleep periods in all participants. Typical sleep periods were defined as beginning between the hours of 8:00 PM to 4:00 AM and ending between the hours of 4:00 AM and 12:00 PM. (**A**) Manhattan plot of SNV associations with total, light, deep, and REM sleep, color coded by sleep phenotype and (**B**) Venn-diagram indicating the number of significant SNVs for total, light, deep, and REM sleep across atypical sleep periods. (**C**) Manhattan plot of SNV associations with total, light, deep, and REM sleep, color coded by sleep phenotype and (**D**) Venn-diagram indicating the number of significant SNVs for total, light, deep, and REM sleep across typical sleep periods.

**Figure S5.** *Identification of SNVs associated with sleep architecture in participants with up to or greater than 90% typical sleep periods.* Mean sleep stage duration was aggregated over all sleep dates in individuals with up to (N = 4,106) or greater than 90% (N = 11,005) typical sleep periods defined as beginning between the hours of 8:00 PM to 4:00 AM and ending between the hours of 4:00 AM and 12:00 PM. (**A**) Histogram showing the distribution of typical sleep period percentage. (**B**) Manhattan plot of SNV associations with total, light, deep, and REM sleep, color coded by sleep phenotype and (**C**) Venn-diagram indicating the number of significant SNVs for total, light, deep, and REM sleep in individuals with ≤ 90% typical sleep periods. (**D**) Manhattan plot of SNV associations with total, light, deep, and REM sleep, color coded by sleep phenotype and (**E**) Venn-diagram indicating the number of significant SNVs for total, light, deep, and REM sleep in individuals with > 90% typical sleep periods.

**Figure S1.**


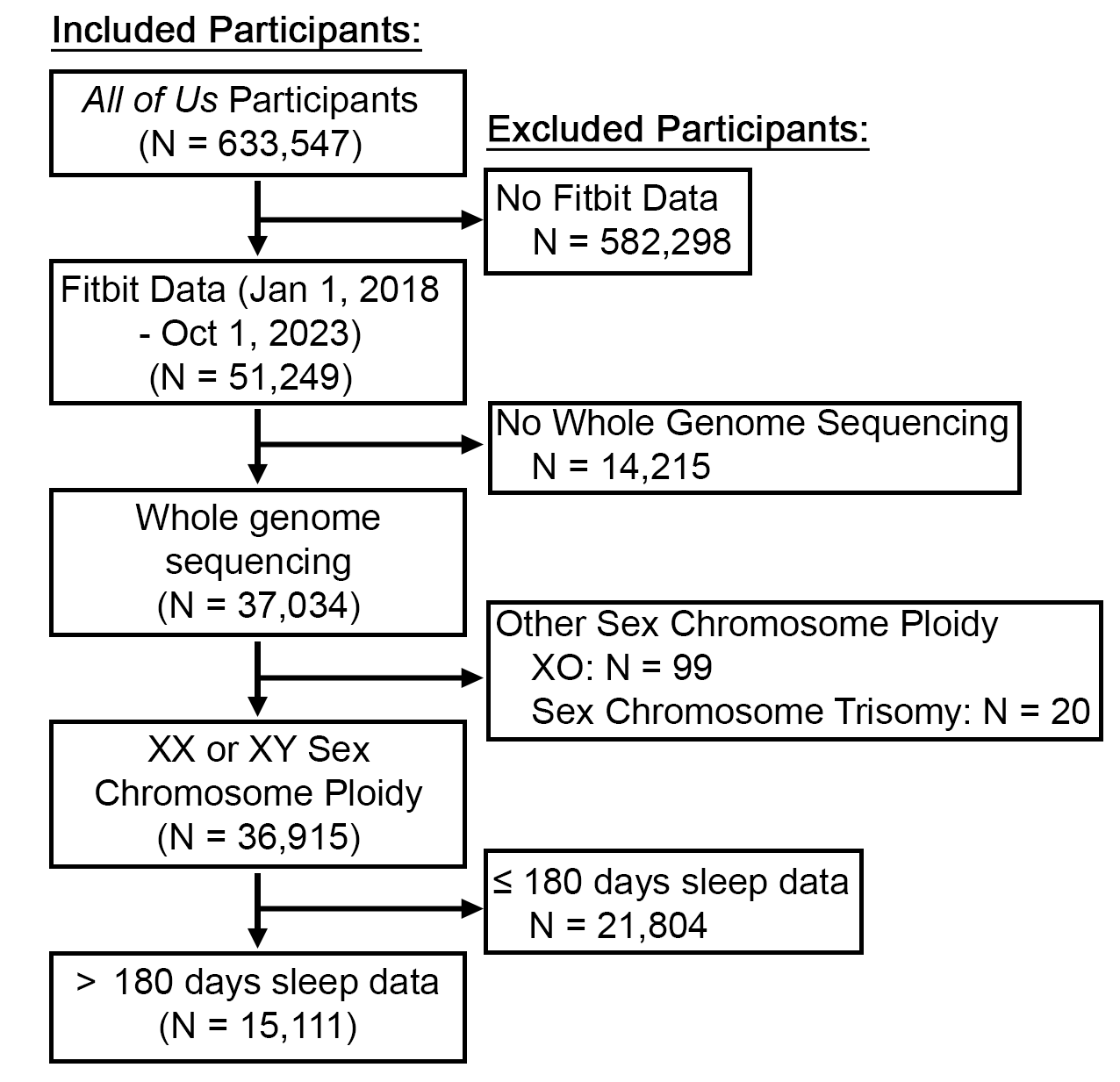


**Figure S2.**


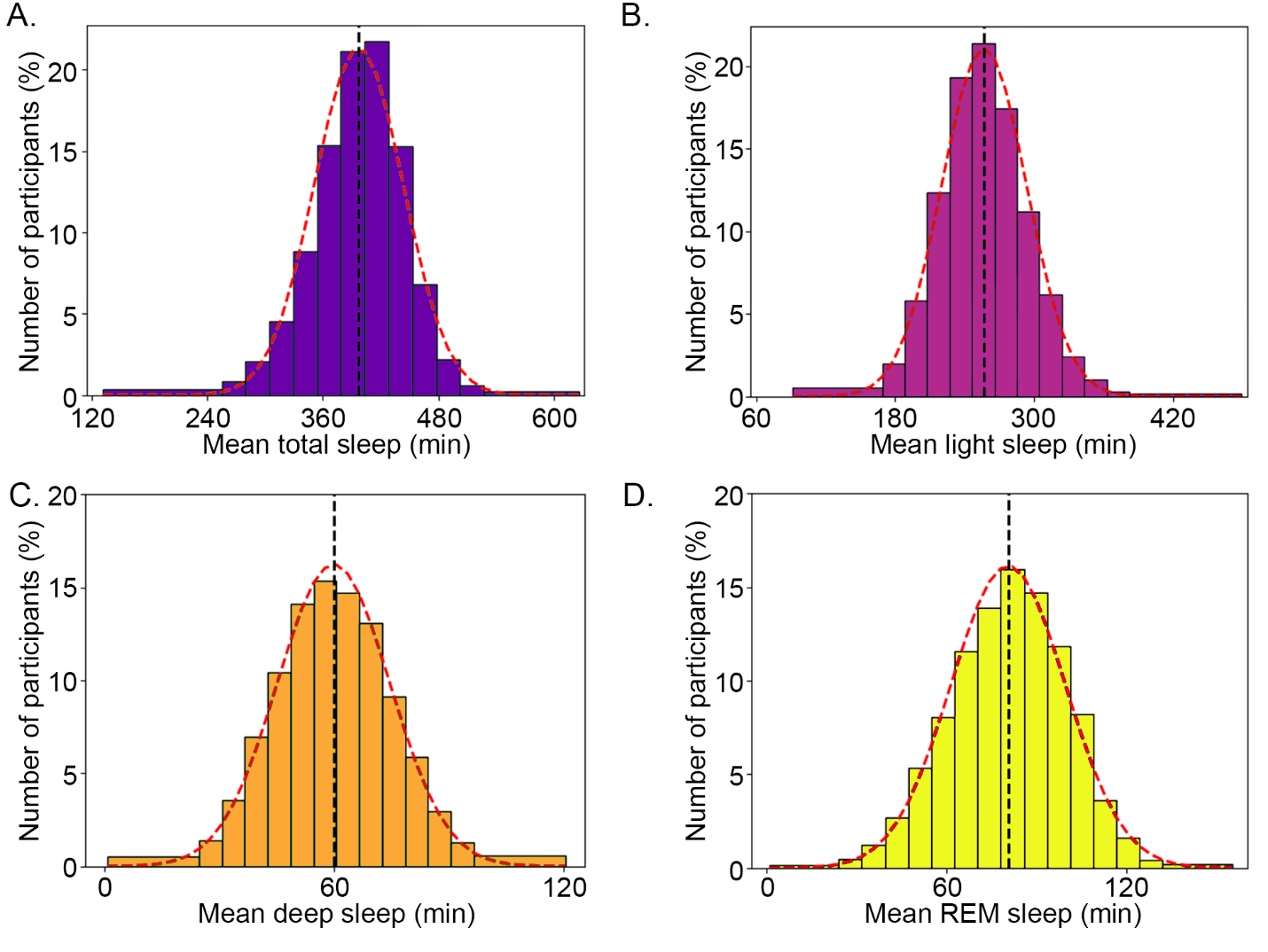


**Figure S3.**


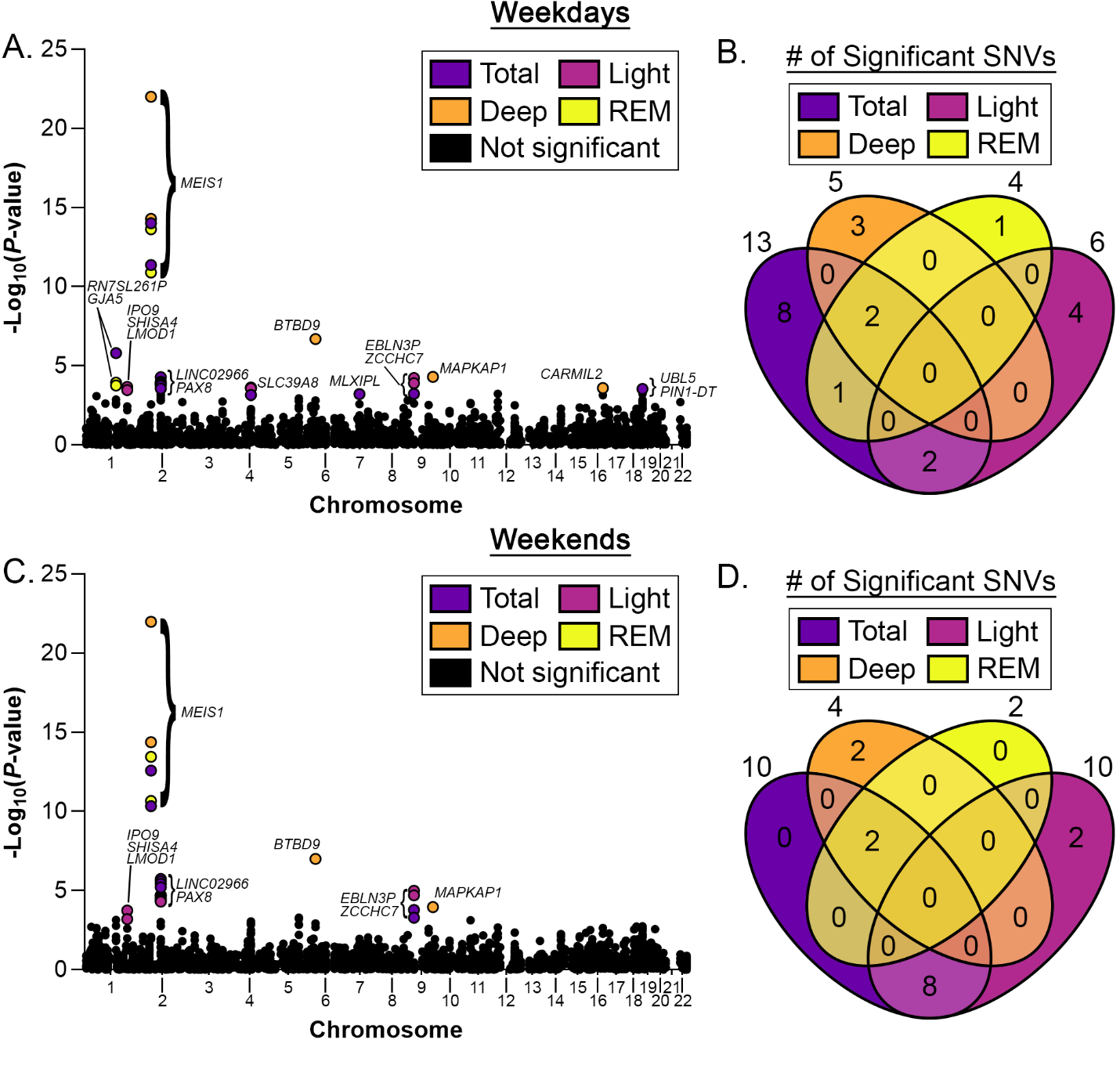


**Figure S4.**


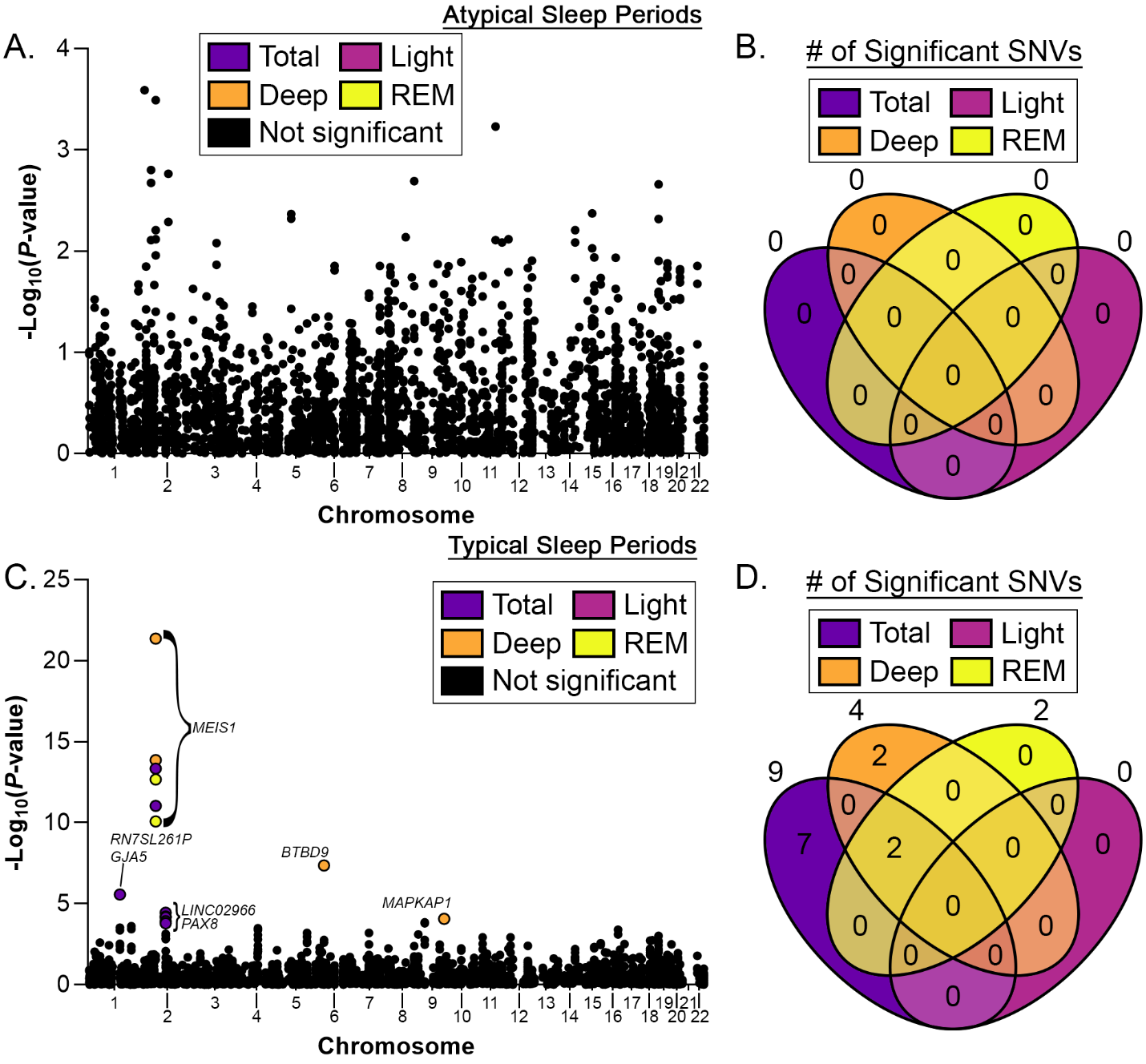


**Figure S5.**


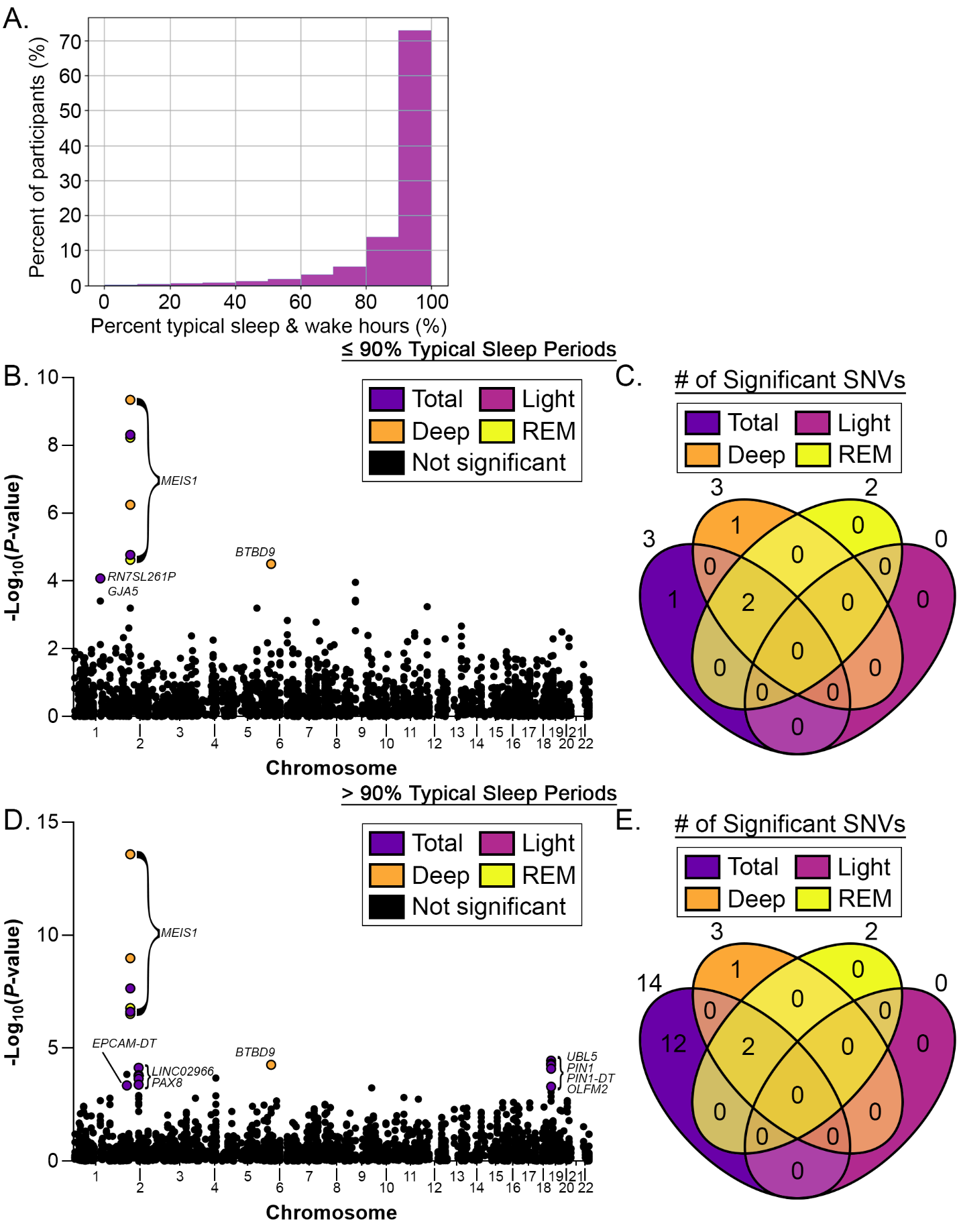

Supplement: Chan_et_al_2025_Supplemental_Figures_zsaf209 [file chan_et_al_2025_supplemental_figures_zsaf209.docx]
